# The Relationship Between Psychedelic Use and Positive Adult Development in Emerging Adulthood: An Integrative Review

**DOI:** 10.1002/brb3.71043

**Published:** 2025-11-14

**Authors:** Jake Payne, Oliver Robinson, Kevin St. Arnaud, Jacob S. Aday, Melissa Warner, Chris Ludlow, Greg Murray

**Affiliations:** ^1^ Centre for Mental Health Swinburne University of Technology Melbourne Victoria Australia; ^2^ School of Human Sciences University of Greenwich London UK; ^3^ Department of Oncology University of Alberta Edmonton Alberta Canada; ^4^ Department of Anesthesiology University of Michigan Ann Arbor Michigan USA

**Keywords:** classic psychedelics, developmental psychology, emerging adulthood, positive adult development

## Abstract

Preliminary evidence suggests that classic psychedelics may exert lasting positive effects on individuals’ mood, cognition, and personality, but little is known about how these effects may differ across the lifespan. Emerging adulthood (18–29) is a critical developmental stage in which individuals transition from dependence on community institutions toward full independence. It is a time when psychedelic experimentation is common, and psychedelics could have distinctive psychological impacts during this phase, although the developmental significance of such effects remains largely untested. This integrative review offers a framework to generate hypotheses regarding possible ways that psychedelic use may positively interact with emerging adult development. Here, we review pathways by which classic psychedelics may facilitate emerging adults’ positive orthogenetic, veridical‐epistemic, eudaimonic, relational, and ethical developmental trajectories through changes in personality trait openness, belief systems, self‐insight, and social concern. Finally, we offer future research directions and discuss key challenges for research into studying the developmental impact of classic psychedelic use in emerging adulthood.

## Graphical abstract

This integrative review explores how classic psychedelic use might broadly be associated with aiding and/or posing risks to emerging adult development along five possible developmental trajectories: orthogenetic, eudaimonic, veridical‐epistemic, relational, and ethical.

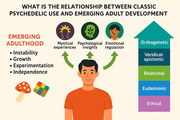

## 1 Introduction

### 1.1 Emerging Adulthood

Emerging adulthood is a theoretical construct that refers to a normative, transitional stage of life that begins around 18 years of age and lasts approximately 10 years, during which individuals engage in developmental processes of discovering their identity, exploring future roles, and assuming the normative tasks of adulthood, while typically deferring commitments such as parenthood (Arnett 2001; Schulenberg et al. 2005). The meaning of adulthood within the term “emerging adulthood” typically includes achievement and assumption of responsibility in education, work, love, friendship, and citizenry (Schulenberg et al. 2004). Emerging adulthood is hypothesized to have appeared as a phenomenon in the West around the 1970s, at which point parenthood and entry into the workforce started to be postponed for the majority. This in turn allowed for a post‐adolescent period of continued personal development and exploration (Arnett 2001). In terms of cross‐cultural validity, while the theory of emerging adulthood was initially developed based on US and European data, supporting research from countries including Pakistan, Indonesia, India, Turkey, and Brazil now evidences that it extends beyond the industrialized West (Choudhury and Raghavan; Dutra‐Thomé and Koller 2019; Numan et al. 2025; Mitra and Arnett 2021). Longitudinal research indicates that emerging adulthood is marked by significant shifts in personality traits—particularly increases in conscientiousness, agreeableness, and emotional stability—and declines in neuroticism relative to adolescence (Roberts and Mroczek 2008). It is also the peak period for the onset of most mood, anxiety, and substance use disorders (Kessler et al. 2007), reflecting both heightened developmental plasticity and vulnerability. During this stage, normative transitions such as leaving home, pursuing higher education or employment, and forming romantic relationships create opportunities for psychological growth but can also precipitate stress, instability, and psychopathology when poorly managed (Wood et al. 2018; Robinson 2015). The negative developmental implications of emerging adult substance use, particularly alcohol and cannabis, have been extensively examined (Schulenberg et al. 2004). Previous developmental research studying the implications of emerging adult substance use has typically emerged from what is known as the pathological drug paradigm (Moore 2008), considering mainly associated harms and not instances of potential constructive use (St. Arnaud 2023).

For some emerging adults, using substances may have an intentional developmental aim, as they may want to experiment with altered states of consciousness in this period before they assume responsibilities that make such experimentation more challenging and potentially problematic (Staff et al. 2010). Currently, little research has studied the potential positive developmental implications of taking classic psychedelic substances, despite their relatively greater use in this phase of life (Johnston et al. 2022). Over the past two decades, there has been a surge in research linking psychedelic use with a myriad of positive psychological constructs, such as increased well‐being, mindfulness, awe, aesthetic experience, and gratitude (Aday et al. 2025, 2024; Jungaberle et al. 2018). Despite these findings, there has been a notable lack of focus on how classic psychedelic substances may potentially interact with emerging adults’ positive developmental trajectories. Integrating empirical knowledge of the distinct opportunities and challenges inherent to this period of life may help counter the risks of psychedelic misuse and maximize positive classic psychedelic instrumentalization during this critical developmental stage (St. Arnaud 2023; Wood et al. 2018).

### 1.2 Classic Psychedelics

Classic psychedelics such as psilocybin, lysergic acid diethylamide (LSD), dimethyltryptamine (DMT), and mescaline are a class of substances distinguished by their action as potent serotonin 2A receptor (5‐HT2A) agonists (Nichols 2016). For brevity, we refer to classic psychedelics simply as “psychedelics” from herein. The term psychedelic combines the Greek words “psyche” (soul or mind) and “dēloun” (to make visible, to reveal). In humans, psychedelic substances alter perception, emotion, and cognition in a dose‐dependent manner (Johnson et al. 2019). Psychedelics are associated with a wide variety of subjective effects including but not limited to mystical‐type experiences, ego‐dissolution, confusion, heightened emotions, and a sense of awe (Nichols 2016). Some psychedelic substances, such as DMT and psilocybin, have been used in indigenous cultures for thousands of years (Fotiou 2019). Today, they are illicit in most countries, although certain psychedelics have gradually become either decriminalized or legalized for medical use in some jurisdictions (Plesa and Petranker 2022).

In the shadow of a surge of interest in the therapeutic potential of psychedelic‐assisted psychotherapy (Schindler and D'Souza 2022), psychedelics are most frequently used outside of research or medical settings, in so‐called naturalistic settings. Controlled experimental and cross‐sectional studies have found sustained positive changes in attitudes, mood, and behavior following administration of high doses of psychedelics with minimal psychological support in nonclinical populations (Griffiths et al. 2018; Griffiths et al. 2006; Schmid and Liechti 2018; Smigielski et al. 2019). Preliminary evidence indicates that classic psychedelics may have the potential to affect psychological personal development in unique ways compared to other substances (Lyvers, and Meester 2012; Deligianni et al. 2023). For example, in a study of 2796 young Swiss men comparing the impact of different substances over time, consciousness alterations induced by psychedelics were reported to have a positive influence on their life but not other substances like cocaine, MDMA, and heroin (Deligianni et al. 2023). This is supported by a retrospective qualitative study by La Torre et al. (2024), which found that a sample of mostly emerging adults reported themes relevant to personal growth in relation to their psychedelic use, including increased insights, greater appreciation for life, improved relationships, and enhanced self‐acceptance.

The outcomes of psychedelic use are influenced by the surrounding context, colloquially known as the “set and setting” (Hartogsohn 2016). “Set” refers to individual factors before and during a psychedelic experience, such as personality, expectancy, and mood, whereas “setting” refers to the physical, social, and cultural environment (Leary et al. 1963). A component of set and setting that has received less attention may be an individual's developmental phase of life. For example, outcomes for older adults may be more connected to existential themes, while outcomes for emerging adults may be related to their future such as career and identity. This is consistent with the conceptualization of psychedelics as “nonspecific amplifiers,” which suggests psychedelics accelerate latent psychological processes and direction of development (Grof 1988). Thus, further research is required to understand how using psychedelics may interact with and potentially amplify broader processes that characterize developmental periods such as emerging adulthood.

### 1.3 Psychedelic Use in Emerging Adulthood

Psychedelics (usually psilocybin or LSD) are most commonly initiated between 18 and 21 years old and are more often consumed during emerging adulthood than at any other stage of life (AIHW 2024; Johnston et al. 2022). In Western countries, between 5.7% and 8% of emerging adults report using psychedelics in the previous year (AIHW 2024; Johnston et al. 2022). In recent years, psychedelic use has appeared to be increasingly socially accepted and culturally embedded for emerging adults (Livne et al. 2022). Indeed, emerging adults (particularly males) appear to have more positive attitudes toward psychedelics than any other age group (Žuljević et al. 2022; Livne et al. 2022). Additionally, various studies (e.g., Donnellan and Lucas 2008) have found that openness to experience and risk‐taking are typically highest in emerging adulthood compared with older adults, which may explain the relatively high usage of psychedelics in emerging adulthood (Terracciano et al. 2008).

Although preliminary, consistent trends have begun emerging suggesting that emerging adults may be uniquely impacted by their use of psychedelics compared to older demographics (Aday et al. 2024, 2021; Izmi et al. 2024). A review by Aday et al. (2021) found that younger individuals may be affected more intensely, both negatively and positively, by psychedelics than older adults. Younger age was found to be more likely to be associated with both adverse effects, such as feelings of dread and impaired cognitive control, and positive effects, such as mystical‐type experiences (Aday et al. 2021). A prospective study by Izmi et al. (2024) compared those near the emerging adult period (i.e., adolescents; 16–24 years) with older adults (>25 years) and found equivalent improvements in psychological well‐being, depression, suicidality, self‐esteem, and emotional stability between the groups, but younger adults reported adverse effects at a higher rate and were more likely to report experiences of paranoia, insanity, fear, and symptoms of hallucinogen‐persisting perception disorder (HPPD). Similar findings have been replicated (Aday et al. 2024; Kettner et al. 2024) and may be partly explained by immature development of the prefrontal cortex (Tamnes et al. 2010) or greater binding potential of 5‐HT2A receptors in younger compared to older adults (Rosier et al. 1996).

From a developmental psychology perspective, just as emerging adults encounter first‐time experiences that mark turning points (i.e., breaking up with a romantic partner or international travel) (McKay et al. 2019), some psychedelic experiences may be a major turning point or what some have termed “pivotal mental states” (Brouwer and Carhart‐Harris 2021). It may be that due to emerging adults’ ongoing brain development, transitional life phase, and limited life experience, emerging adults’ psychedelic experiences may be more significant turning points than later in life. Psychedelic experiences that involve mystical‐type experiences (Griffiths et al. 2008), emotional breakthroughs (Roseman et al. 2019), psychological insights (Davis. et al. 2021), feelings of self‐transcendence (St. Arnaud and Sharpe 2023b), feelings of openness (Smigielski et al. 2019), and connectedness to one's surroundings (Kettner et al. 2019) may constitute significant positive turning points for some emerging adults’ ongoing development. Such acute effects of psychedelic experiences have been identified as psychological mechanisms of change associated with improved long‐term well‐being (Aday et al. 2020; Izmi et al. 2024) and, we hypothesize, could contribute positively to emerging adult development.

In addition to potentially aiding development, there is some evidence pointing to the potential for psychedelic use to be involved in negative developmental trajectories. For example, psychedelic use can trigger latent psychotisis spectrum disorders (Dos Santos et al. 2017; Cerón Tapia et al. 2022) or lead to the development of hallucinogen use disorder and HPPD, all of which are most prevalent during emerging adulthood (APA 2022). Furthermore, it is possible that less researched outcomes of psychedelic use such as ego inflation (Hendin and Penn 2021; Masters and Houston 2000), false insights (McGovern et al. 2024), and social disconnection (Bremler et al. 2023) could potentially hinder emerging adult development. These areas warrant further research and are discussed further at the end of this article. A key focus for future research into potential developmental impacts of psychedelic use is the question of what factors might influence outcomes from psychedelic use toward *positive* as opposed to negative or mixed developmental trajectories. Preliminary studies indicate that an individual's intention for psychedelic use may play a role in the outcome of psychedelic use (Carhart‐Harris et al. 2018). For instance, psychedelic use motivated by self‐expansion—such as for personal growth or self‐reflection—has been linked to beneficial outcomes (St. Arnaud 2021), whereas use for recreation or to escape negative emotions has not (St. Arnaud and Sharpe 2023a). Further research is required to understand how an individual's age, personality, developmental period, attachment style, patterns of psychedelic use, and types of psychedelic experiences moderate outcomes. Next, we outline some tentative pathways through which psychedelic use may interact with a broad number of trajectories of positive adult development in emerging adulthood (Robinson 2020) with the aim to generate hypotheses and encourage future research.

### 1.4 Positive Adult Development in Emerging Adulthood

Positive adult development in emerging adults is complex and multifaceted; consequently, there are a range of theories and conceptual frameworks for understanding these processes (Erikson 1968; Levinson 1986). Emerging adults inevitably face the reality of development as an outcome of trade‐offs, compromises, and managing conflicts (Luke and Redekop 2014). The framework drawn on in this review was developed by Oliver Robinson (Robinson et al. 2015, 2013; Robinson et al. 2018; Robinson 2019; Robinson 2020). Robinson's (2020) framework for positive adult development includes five overarching trajectories: orthogenetic, veridical‐epistemic, eudaimonic, ethical, and relational‐reproductive. For the hypothesis‐generating purposes of this review, Robinson's broad multifactorial approach is a strength, as is the theorist's recognition that different developmental trajectories can be dialectically juxtaposed or conflicting at times. These trajectories are interactive and mutually formative, but can be separated out for analytical purposes (Robinson 2020).

**FIGURE 1:**
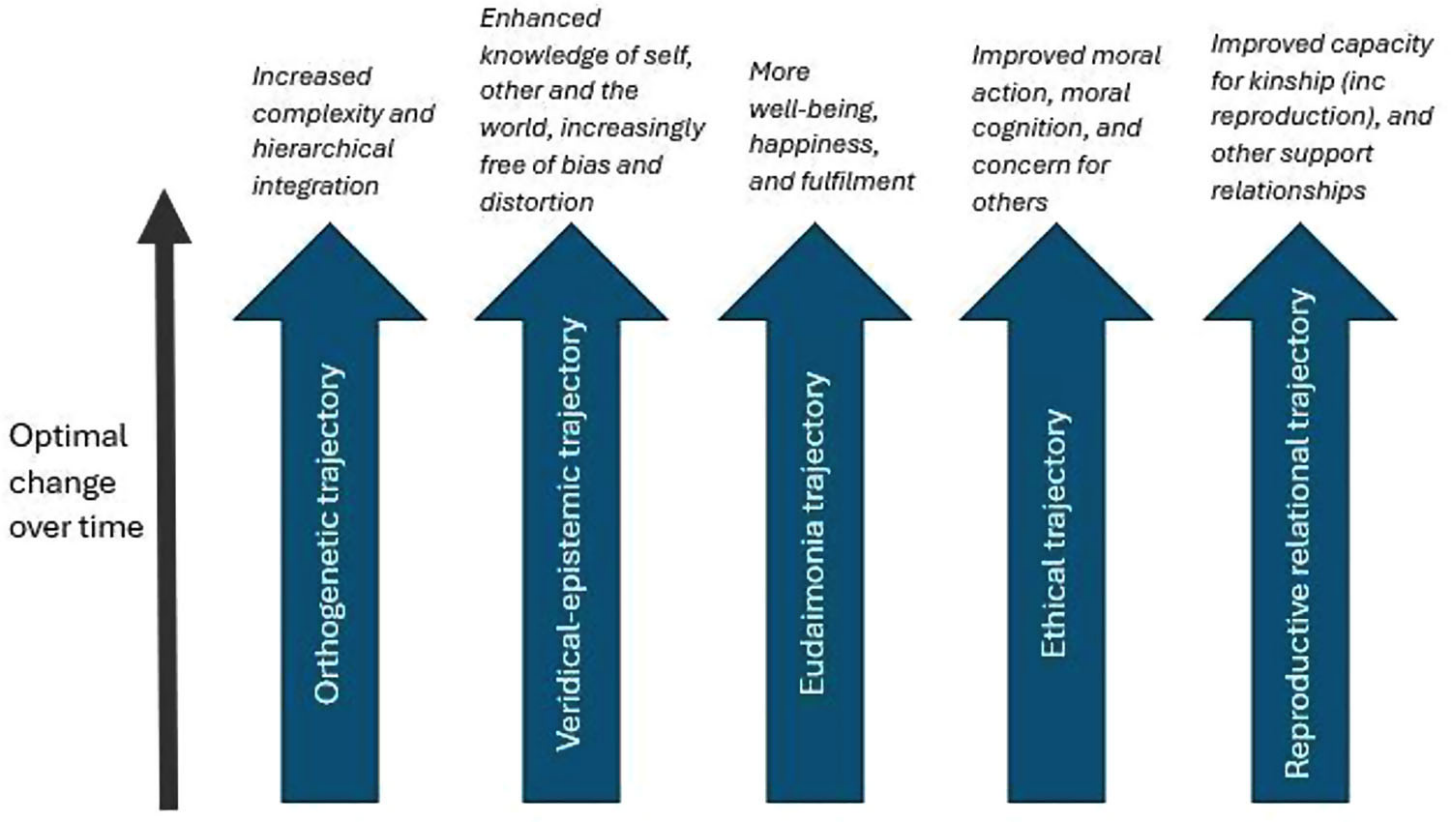
Robinson's (2020) five trajectories of positive adult development.

For Robinson, the *orthogenetic trajectory* of adult development refers to increases in integrated complexity over time by way of differentiation, articulation of elements into parts, and hierarchical integration of those parts into systems (Robinson 2020). For an emerging adult, this may entail managing the complexity of being responsible for both themselves and newly adopted responsibilities, such as living out of home, education, or work (King and Kitchener 2015). Linked to this, but distinct, is the *veridical‐epistemic trajectory*, which involves progressive shifts toward a less biased and distorted view of self, others, and the environment over time (Robinson 2020). For an emerging adult, this may involve furthering processes such as individuation, wherein they are psychologically differentiating themselves from their family and others (Koepke and Denissen 2012). The *eudaimonic* trajectory refers to ways in which a person develops toward flourishing or self‐actualization, and involves a sense of personal growth, purpose, meaning, emotional well‐being, and self‐acceptance (Robinson 2020). This may involve the sense of meaning and purpose gained from realizing a rewarding career or achieving a life goal for emerging adults (Proctor et al. 2015). The *ethical* trajectory of positive adult development refers to increases in moral concern for, and ethical actions toward, others (Robinson 2020). For emerging adults, this may involve gaining a sense of personal or collective identity and purpose through supporting or acting for a social cause (Lapsley and Hardy 2017). Finally, the *relational‐reproductive* trajectory focuses on increasing social connectedness and caring for the next generation by way of having children or other acts of generativity (Robinson 2020). This may involve becoming a parent, experiencing their first committed relationship, or developing a sense of belonging to a social group for emerging adults (Cashen and Grotevant 2020). Figure 1 displays Robinson's (2020) trajectories; next, we review plausible pathways by which psychedelic use may positively impact each of these.

## 2 Psychedelics and Positive Emerging Adult Development

### 2.1 Orthogenetic Development

According to Commons and Ross's (2008) Model of Hierarchical Complexity (MHC), development in postformal cognition is an example of increased complexity and hierarchical integration. The concept of postformal cognition expands upon Piaget's theory of cognitive development and thus recognizes advanced, or *postformal*, cognitive processes in adulthood (Commons and Richards 2003). Postformal development is an individual's adaptive style, search for meaning, intelligence, choices, and processes that change to accommodate a more complex way of perceiving truth and meaning (Griffin et al. 2009). There are various conceptual models of postformal thought, but each theory has several components in common: the ability to identify, understand, and integrate contradictory information, the ability to incorporate information into larger wholes or meta‐frameworks, and the ability to recognize that knowledge is relativistic. Postformal stages of cognitive development *may* (though are not guaranteed to) emerge for the first time in emerging adulthood (Sinnott 1998).

Griffin et al. (2009) found a significant positive relationship between postformal cognition and trait openness to experience. This is perhaps unsurprising, given that postformal thought involves multiple views of reality co‐existing and might support a person to flexibly integrate new ideas, thoughts, feelings, and aesthetic experiences into schemas to create a more expansive view of reality. Given that the use of classic psychedelics is associated with increases in trait openness to experience (Erritzoe et al. 2019) and cognitive flexibility (Doss et al. 2021), they may positively contribute to the development of postformal cognition. Furthermore, psychedelics have been shown to induce complex and novel aesthetic experience (Aday et al. 2025), such as intricate visions that have symbolic meanings and paradoxical implications (Kometer and Vollenweider 2018). Such experiences may potentially broaden an emerging adults’ perception of reality and capacity for perspective taking, which may in turn promote the development of multiple views of reality, thus facilitating postformal development.

Another pathway where psychedelics may positively impact orthogenetic development is through changes to belief systems. According to Marko (2011), development progresses when persistent inconsistencies appear and cannot be incorporated into the individual's current system of beliefs. In these instances, reorganization to a higher order of integration occurs (Marko 2011). It is possible for changes of this type to take place through a new awareness, a new feeling about life, or suddenly seeing something previously hidden. Enabling factors, known as *developmental pacers* or *facilitative experiences*, may move development incrementally forward or through a bolt of sudden awareness (Marko 2011). Developmental pacers/facilitative experiences are catalysts that provide the experience, knowledge, or understanding required to release the individual from his or her existing paradigm and facilitate vertical development.

Research conducted by Marko (2011) found that although facilitative experiences can occur in normal waking consciousness, they seem to happen more often in *altered states of consciousness*, including those evoked by the use of psychedelics. Marko (2011) suggests that the control demanded by the ego is less able to discredit or disregard data inconsistent with its established beliefs when in an altered state, thus prompting accommodation and reorganization of current beliefs. This is consistent with the Relaxed Beliefs Under pSychedelics (*REBUS*) model of psychedelic function proposed by Carhart‐Harris and Friston (2010). The model proposes that psychedelics decrease the power of prior, higher order beliefs and thus allow for re‐organization of lower level behaviors and beliefs. In other words, they remove fixed certainties and bring openness to what is novel. Indeed, psychedelic experiences have been found to alter belief systems (Timmermann et al. 2021; Amada et al. 2020). Amada et al. (2020) proposed that changes to self‐narrative are at the center of psychedelics’ therapeutic and transformative potential. They gathered 418 retrospective reports from healthy individuals of changes to self‐narrative that were believed to be instigated by their psychedelic experience(s). Their results revealed themes including decentered introspection, greater access to self‐knowledge, positive shifts in self‐evaluation, greater psychological and behavioral autonomy, as well as enhanced connectedness with others, supporting their proposition of the role of psychedelics in restructuring self‐narratives.

A notable belief system change induced by psychedelics regards changes in spirituality (Letheby and Gerrans 2017). Emerging adults explore beliefs through socialization agents, such as peers (e.g., friends, romantic partners, classmates, and coworkers) who may hold more religiously and spiritually diverse views than their own. Psychedelic experiences can be deeply personally meaningful and hold pronounced spiritual significance (Griffiths et al. 2006) and have the potential to alter metaphysical beliefs (Timmermann et al. 2021). For some emerging adults, a psychedelic experience may be their first experience of a particularly life‐changing event akin to a significant loss, like the death of a loved one, or gain, such as the birth of a child. These experiences may be a type of socialization agent that introduces them to spirituality (Letheby 2021; Levenson et al. 2005). Thus, it is arguable that the use of psychedelics in emerging adulthood may facilitate the adoption of more spiritual beliefs. Indeed, a recent study of psilocybin‐assisted psychotherapy for alcohol use disorder found that participants reported spiritual insights, beatific visions, and communion with the Divine, which they considered fundamental to positive treatment outcomes (Podrebarac et al. 2021). Importantly, scholars suggest that among emerging adults, spirituality can offer opportunities for constructive self‐reflection that may lead to short‐ and long‐term positive outcomes (Regnerus 2003; Hoover 2016). For example, it has been suggested that spirituality among emerging adults can protect against engaging in “risk behaviors,” such as substance use, illegal and deviant conduct, and sexual activity (Yonker et al. 2012), that often pose risks to health and well‐being. Nonetheless, little is known about whether the form of spirituality fostered by psychedelics can have beneficial implications for the overall welfare of emerging adults (Schutt et al. 2024).

Another pathway we propose that psychedelic use may positively impact cognitive and hierarchical integration is through ego development. In Loevinger's (1976) theory of ego development, the *ego* is an integrative schema through which people make meaning. It functions as an orchestrator of one's worldview—the traits, concerns, and self‐narratives that organize one's personality and goal‐directed activity (Westenberg and Gjerde 1999). Ego development is marked by nine different ego stages, each of which designates a new way of organizing experience (Loevinger 2014). Research has demonstrated that most US adults stabilize at the fifth, or *Self‐aware* ego stage (Cohn &Westenberg. 2004). Cohn and Westernberg's meta‐analysis suggests that gains in ego level are frequent in early adolescence, slower in late adolescence, and infrequent in emerging adulthood and beyond. Most emerging adults (and adults more generally) do not develop beyond the Self‐aware stage, which suggests a “ceiling” effect in development. Westenberg and Gjerde (1999) speculate that contemporary Western society stimulates growth toward the Self‐aware level but does not provide the necessary experiences or contexts needed to challenge an individual's mental frame beyond this level. Indeed, Loevinger (1976) sees the transition beyond the Self‐aware stage as a major shift not explainable in terms of *external* pacers/facilitative agents (e.g., educational or interpersonal contexts). Instead, advanced development may be based on *internal* pacers/facilitative agents (Westenberg and Gjerde 1999). Manners et al. (2004) reported that internal facilitative experiences that are disequilibrating/challenging, personally meaningful, and emotionally charged seem to facilitate advanced ego development. Similarly, Helson and Roberts (1994) point to the importance of an optimal level of *accommodative challenge* to facilitate development. An adult life high in accommodative challenges would be one high in both positive and negative disruptions, such as finding or losing a partner, success in or loss of employment, or the death of a loved one. Given that the use of psychedelic substances may prompt experiences that destabilize and challenge previously held beliefs (Carhat‐Harris and Friston 2010; Johnstad 2021), are deeply personally meaningful (Griffiths et al. 2006), and are emotionally charged (Roseman et al. 2019), they may be associated with processes that could theoretically support the development of postformal cognition, though direct evidence for this remains limited.

### 2.2 Eudaimonic Development

Eudaimonic development involves growth toward full realization of one's potential, optimal well‐being, or flourishing, and self‐actualization (Ryan and Deci 2001; Waterman 2008). Research indicates that successfully establishing personally meaningful identity commitments through a process of exploration can provide the foundation for eudaimonic development in the period of emerging adulthood (Waterman 2008). Valde (1996) differentiated between “open” and “closed” identity achievement among emerging adults. Those with *optimal* identity achievement appear to remain open to re‐exploring their goals, beliefs, and values throughout adulthood, while still committing to life tasks and roles, and demonstrate higher levels of self‐actualization. Tesch and Cameron (1987) argue that openness to experience might facilitate this ongoing exploration of identity, thus facilitating an “open‐achieved” identity in those who have made commitments to a given life structure. Similarly, other lines of research have demonstrated that openness to experience is among the most important predictors of eudaimonic development (Levenson et al. 2005; Staudinger and Bowen 2010). Given the capacity of psychedelics to increase trait openness to experience (Erritzoe et al. 2019), promote self‐insight (Davis et al. 2021), and facilitate cognitive flexibility (Davis et al. 2022; Doss et al. 2021), it is plausible that psychedelic use may catalyze meaningful identity exploration and thus aid eudaimonic development in emerging adulthood.

Eudaimonic development and positive identity growth more broadly have also been shown to be facilitated by processing and working through conflicts and painful emotional experiences (Pals 2015; Whitehead and Bates 2016). As such, the use of psychedelics may lead to positive shifts in the eudaimonic developmental trajectory via psychedelic‐induced *emotional breakthroughs* (Roseman et al. 2019). An emotional breakthrough involves facing thoughts, images, or memories that have a painful emotional valence and then “breaking through” the experience to discover a sense of resolution or relief (Roseman et al. 2018). Emotional breakthroughs during psychedelic experiences are thus akin to catharsis, wherein processing a difficult inner conflict results in a sense of release (Roseman et al. 2019). Importantly, emotional breakthroughs during psychedelic experiences have been found to predict improvements in well‐being (Roseman et al. 2018). As emerging adults are in the process of developing emotional autonomy (Cole et al. 2004) and many have difficulties in expressing emotion (Ryan and Lynch 1989), psychedelic‐induced emotional breakthroughs may aid their overall eudaimonic development at this stage of life.

It has been proposed that an integral component of eudaimonic well‐being is self‐determination (Deci and Ryan 2008). Ryan and Deci (2000) view self‐determination as a motivational orientation defined by being both intrinsically and autonomously motivated (Deci and Ryan 2008). It includes three components: competence (i.e., confidence and a sense of self‐efficacy), autonomy (i.e., approval of one's actions), and interpersonal relatedness. Teixeira et al. (2021) proposed that these three facets may be positively influenced by psychedelics, citing qualitative accounts of participants in psilocybin open‐label research trials for depression (Watts et al. 2017), alcohol cessation (Nielson et al. 2018), and smoking cessation (Noorani et al. 2018). With respect to competence, Nielson et al. (2018) found that participants qualitatively reported themes from their responses of increased confidence, motivation, resolve, and commitment to change as related to their psilocybin experiences. Similarly, Watts et al. (2017) reported that participants felt more confident, resilient, and effective on account of their drug experiences. With respect to autonomy, Noorani et al. (2018) reported that participants described insights into self‐identity and authenticity on account of their psilocybin sessions, while Watts et al. (2017) reported participants felt more in line with their internal needs and inherent worth. In terms of relatedness, Noorani et al. (2018) noted that psychedelic sessions left participants with a sense of interconnectedness and increases in prosocial behavior. Collectively, these findings suggest a potential for psychedelic use to align with factors theorized to underpin eudaimonic development.

### 2.3 Veridical‐Epistemic Development

Veridical‐epistemic development involves developing a more accurate and unbiased sense of self, other, and reality at large by overcoming cognitive biases and distorting defense mechanisms (Robinson 2020). Indeed, many studies have shown that developing accurate personal insight into one's thoughts, feelings, and behaviors is conducive to mental well‐being (Lyke 2009). Interestingly, self‐insight has been shown to be a common, if not fundamental, property of the psychedelic experiences (see Peill et al. [2022] for an in‐depth overview). For example, Amazonian cultures that utilize psychedelics refer to the plants used in the psychedelic Ayahuasca brew as “teachers” (McKenna 1991). This phenomenon is sometimes referred to as *psychedelic‐catalyzed insight* (PCI) (Kugel et al. 2025) and can be defined as sudden realizations about oneself, a relationship, or an aspect of, or a problem in, one's life (Davis et al. 2021). Several studies have found PCIs to be commonly reported during and after high‐dose psychedelic experiences (Bogenschutz and Forcehimes 2017; Watts et al. 2017). Peill et al. (2022) surveyed 886 participants before and after a psychedelic (74% psilocybin) experience and found that more than 90% reported some degree of personal insight. Clinical studies of psychedelics also suggest that psychological insight correlates with therapeutic benefits (Roseman et al. 2018; Garcia‐Romeu et al. 2020). Numerous studies suggest that psychedelic‐catalyzed insights are associated with greater self‐awareness and understanding (Watts et al. 2017; Noorani et al. 2018; Amada et al. 2020). For example, Watts et al. (2017), Noorani et al. (2018), and Amada et al. (2020) reported that psychedelic participants described PCIs related to identity (e.g., revealing knowledge about their true self) and self‐understanding. Likewise, it is not uncommon for individuals to have PCIs that are particularly relevant to themes found in emerging adulthood, such as relationships, career, and life goals, such as “Awareness of my life purpose, goals, and/or priorities,” “Discovered new feelings or perspectives about significant relationships in my life,” and “ I discovered new insights about my work or career” (Davis et al. 2021).

Veridical‐epistemic development also involves cultivating self‐knowledge and observing oneself, others, and the world more accurately (Robinson 2020), capacities supported by metacognitive development (Bernstein et al. 2015). Metacognition, a term introduced by developmental psychologist John Flavell (1979), refers to the awareness and regulation of one's own cognitive processes. Flavell famously described metacognition as “thinking about thinking” (Flavell 1979), emphasizing its foundational role in self‐awareness and cognitive regulation. Over the decades, the concept has evolved to encompass various skills and capacities essential for personal development and psychological well‐being (Lai 2011). Developing metacognitive skills supports emotional regulation and mentalizing other minds, enhances problem‐solving abilities, and promotes goal‐setting and achievement (Schraw 2013). Recently, metacognitive models of mindfulness have evolved to integrate these principles, highlighting how such practices can be essential tools in fostering self‐awareness and cognitive flexibility (Jankowski and Holas 2014). Psychedelics have been shown to increase components of mindfulness by enhancing present‐moment awareness, promoting nonjudgmental acceptance of experiences, and facilitating greater cognitive flexibility and emotional regulation (Kähönen 2023; Murphy‐Beiner and Soar 2020).

The two foundational components of metacognition are (1) monitoring and (2) regulating cognition (Brown and Elliot 2016). Monitoring involves the capacity to observe mental arisings or mental objects as representations rather than reality. Regulation, also known as control in the literature, is the capacity to attenuate cognitive and emotional operations for personal optimization (Lai 2011). Regulation is exemplified by processes such as deautomatization, decentering, and re‐perceiving of mindfulness (Shapiro et al. 2006; Bernstein et al. 2015), as well as reality testing and the development of a “metacognitive mode” in metacognitive therapy (Batmaz 2014). The metacognitive mode promotes the ability to distinguish between thoughts and reality, helping individuals recognize that their thoughts are merely mental events rather than direct reflections of the external world. Throughout clinical psychological theories, a metacognitively mindful, decentered, and flexible self‐concept plays a central role in well‐being (Bernstein et al. 2015) and the development of wisdom (Grossman 2015). Brown and Elliot (2016) expanded the definition of metacognition to include postformal skills, including the capacity to develop multiple perspectives, overcome bias, engage in meaning‐making, and even attain non‐dual awareness. Psychedelics can evoke non‐dual experiences, and clinical trial participants frequently report their psychedelic experiences among the most meaningful in their lives, indicating psychedelics may generate transient experiences in emerging adults associated with higher order metacognitive skills (Dowie and Tempone‐Wiltshire 2023).

One of the fundamental biases human cognition faces is the appearance–reality distinction, the metacognitive awareness that experiences of reality are representations of reality (Warnell and Redcay 2019). A lack of awareness of this distinction can hinder personal growth and self‐insight, as individuals cling to inaccurate self‐concepts and fixed beliefs about their thoughts, feelings, and behaviors. John Flavel, who coined the term “metacognition,” described the appearance–reality distinction as the knowledge that “objects or events can be represented in different ways by the same person and by different people” (Flavell 1986). Psychedelic experiences, by the very nature of the phenomenal experience, bring into focus the appearance–reality distinction, from the alterations to the visual world to the boundaries between self, other, and nature (Blacker 2023, 45). The appearance–reality distinction invites an awareness of the limitations of knowledge, the fallibility of subjectivity, the potential for bias, and the value of epistemic humility, all of which are advanced metacognitive skills (Brown and Elliot 2016) and key components of wisdom (Grossman 2015). This capacity of psychedelics to bring into focus the appearance–reality distinction (Blacker 2023, 45), increase aspects of mindfulness (Kähönen 2023), and generate experiences associated with higher order metacognitive views (Dowie and Tempone‐Wiltshire 2023) indicates that psychedelic experience could support the development of metacognitive skills in young adults.

Taken together, the intersection of psychedelics, insight, and metacognition offers a promising avenue for fostering veridical‐epistemic development. By challenging the appearance–reality distinction (Blacker 2023, 91), evoking metacognitive experiences (Dowie and Tempone‐Wiltshire 2023), and enhancing mindfulness (Kähönen 2023; Payne et al. 2021) and psychological insights (Davis et al. 2021), psychedelics can support the development of a more accurate and flexible understanding of oneself and the world. This, in turn, can lead to greater emotional regulation, cognitive flexibility, and overall well‐being (Lyke 2009). Thus, it is reasonable to hypothesize that psychedelic use, under the right conditions, may significantly contribute to veridical‐epistemic development, particularly in emerging adults who are navigating critical developmental tasks related to identity, relationships, and life goals.

An important caveat when determining optimal development on the veridical epistemic trajectory is that individuals with differing worldviews may disagree on what constitutes enhanced accurate knowledge about reality. For example, lifetime psychedelic use has been found to be associated with religious nonaffiliation, which would be seen as negative from a traditional religious perspective but positive from a spiritual but not religious standpoint (Cherniak and Granqvist 2025). In sum, determining what is or is not an accurate representation of reality requires a consensus ontology—that is, an agreement on what reality is—and this itself undergoes considerable changes for many who work with psychedelics (Argyri et al. 2025). Ultimately, this requires any individual to situate and integrate their own developing episteme within cultural and academic discourses (for further discussion, see Sjöstedt‐Hughes 2023).

### 2.4 Relational‐Reproductive Development

Forming intimate relationships is a crucial developmental task that often occurs during emerging adulthood, and successful completion of this task is associated with lower psychological distress and greater well‐being (Gómez‐López et al. 2019; Holder 2012). A large body of research suggests that psychedelic experiences can meaningfully impact the way individuals relate to each other (see Preller and Vollenweider [2019] for a review). For example, Neubert et al. (2024) conducted semistructured interviews with six emerging adult couples aged between 19 and 29 years about their shared psychedelic experiences. They found that the couples described experiencing *psychedelic intimacy*, which involved greater undivided attention and positive regard for the other, feeling more connected and present with the other, and an ability to speak more clearly about important things to each other. Similarly, Barba et al. (2024) found preliminary quantitative evidence to suggest psychedelic use is associated with improvements in several facets of sexual functioning in naturalistic and clinical populations. These findings are supported by cross‐sectional studies that have found associations between lifetime psychedelic use and themes relevant to relationships, such as reduced intimate partner violence (Thiessen et al. 2018) and higher emotional regulation (Walsh et al. 2016) compared to non‐psychedelic users.

Psychedelic use in emerging adults may impact not only their intimate relationships but also their platonic relationships (Pokorny et al. 2017). Forming platonic like‐minded and supportive friendships is another important developmental task of emerging adulthood (Barry and Madsen 2010). A systematic review by Pezirkianidis et al. (2023) underscored the relationship between high‐quality peer relationships and subjective happiness and well‐being in emerging adults. Research suggests that a single psychedelic experience may increase prosocial behavior such as enhanced empathy and emotional connectivity with others (Preller and Vollenweider 2019). For example, an observational study by Pokorny et al. (2017) with 32 emerging adults who were administered a high dose of psilocybin found an increase in emotional empathy 10 days after. A naturalistic study by Newson et al. (2021) (*M* = 30.51 years) found that when psychedelics are used in conjunction with dancing at a rave, awe‐inspiring psychedelic experiences were associated with bonding with other ravers and prosocial behavior. Similarly, while not utilizing an explicitly emerging adult sample, two double‐blind studies (Griffiths et al. 2008, Griffiths et al. 2011) administering psilocybin reported that participants sustained positive changes in relationships with others and sensitivity to others’ needs 14 months after the psychedelic experience. This is further supported by Noorani et al. (2018), who administered psilocybin for smoking cessation and found that the majority of participants reported an increase in prosocial behavior. Moreover, a prospective longitudinal study by Nayak et al. (2025) following a planned psilocybin experience found an increase in mentalization, which is theorized to play a role in maintaining high‐quality friendships (Güroğlu 2022). These preliminary findings raise the possibility that psychedelic experiences could influence factors relevant to relational development.

### 2.5 Ethical Development

For the first time in their lives, emerging adults are asserting themselves to play a more dynamic part in their society (Arnett 2014). Through engagement in solving social issues, emerging adults can develop a sense of personal identity that is meaningful as it involves connecting to historical movements (Youniss, and Yates 1997). A significant aspect of psychological development is situating oneself within broader systems, including relationships with their community and the broader world (Schulenberg et al. 2004). Many emerging adults maintain that greater consideration for others is an important element of maturation into adulthood (Mayseless and Scharf 2003). Studies have found associations between psychedelic use and enhanced concern for social issues and encourage more active citizenship (Paterniti et al. 2022). For example, there appears to be an association between psychedelic use and pro‐environmental values (Aday et al. 2025; Kettner et al. 2019). Moreover, three double‐blind studies (Griffiths et al. 2008, Griffiths et al. 2018, 2011) administering psilocybin found that participants attributed to the psychedelic experience sustained positive changes in social concerns at both 1 and 14 months follow‐up points. Likewise, observational studies by Noorani et al. (2018) and Schmid and Liechti (2018), which administered psilocybin and LSD, respectively, found that participants reported increased altruism, social concern, and pro‐social behavior up to 30 months following the experience.

Self‐transcendent states, which involve a temporarily decreased awareness of self and concomitant feelings of connectedness, belonging, and unity (Yaden et al. 2017), may help explain the ethical developmental potential of psychedelics. Self‐transcendent states are believed to exist on a spectrum of intensity described as the unitary continuum (Newberg and d'Aquili 2000). At the low end of the spectrum are states such as mindful awareness and flow, whereas in the midrange fall the self‐transcendent emotions, such as awe. At the furthest end lies the mystical experience, which, as a particularly potent self‐transcendent state, usually involves complete self‐dissolution, leaving the person feeling a sense of unity with something greater than their personal self (Yaden et al. 2017).

Kahonen (2023) argues that self‐transcendent experiences reduce egocentric evaluative biases and provide enhanced “epistemic access,” as such experiences are beyond any personal viewpoint (2), thus advancing development on both the veridical‐epistemic and ethical trajectories. Koplowitz's (1984) model of unitary cognition may also help explain the potential developmental benefits of psychedelic‐induced self‐transcendent experiences. According to this theory, as thinking becomes more developed, the perceived boundaries between concepts or ideas become less rigid. Unitary cognition allows comprehension of wholes, the oneness of perceived opposites, and connections between parts. Consequently, unitary cognition serves to organize and bring experiences and contents of thought into a greater sense of connection, coherence, and relation to one another (Commons and Richards 2003). Correspondingly, themes of unity, oneness, and connectedness have been discussed extensively in the psychedelic literature (McCulloch et al. 2022). Psychedelic experiences often entail a sense of connectedness with one's own senses, body, and emotions; with friends, family, and community; and with nature, the living world, global humanity, and whatever is perceived as Divine or Sacred (Watts et al. 2017). Similarly, psychedelic users often report self‐transcendent experiences in which one's subjective sense of self fades into an experience of unity with other people or one's surroundings, involving the dissolution of boundaries between the sense of self and other (Yaden et al. 2017).

Hendricks (2018) suggested that experiencing awe is a plausible mechanism by which psychedelics produce their beneficial effects. Awe is a feeling of wonder experienced when facing something vast and beyond understanding or comprehension (Keltner and Haidt 2003). It is the prototypical self‐transcendent emotion, involving a deep, experiential awareness of one's smallness and a concomitant sense of connection with a perspective greater than oneself (Piff et al. 2015). Thus, feelings of awe challenge one's sense of being a separate self, generating expansive feelings of connection, unity, and being part of a vast and greater whole, which may contribute to its beneficial effects (Piff et al. 2015). St. Arnaud and Sharpe (2023b) found that psychedelic‐facilitated experiences of awe predicted both personality adjustment and personality growth in adulthood. Importantly, only individuals who deliberated and sought to reflect upon and integrate their psychedelic experiences into daily life demonstrated a predictive relationship between their use and positive development. Moreover, most participants in St. Arnaud and Sharpe's (2023b) study were between 18 and 34, suggesting the findings may have relevance to emerging adults. Similarly, in a prospective, naturalistic study with ayahuasca, Aday et al. (2025) found that experiences of awe during participants’ ayahuasca ceremonies predicted lasting increases in gratitude and nature relatedness 1 week and 1 month later. Collectively, these findings indicate that psychedelic use and experiences might play a role in enhancing ethical development in some emerging adults.

## 3 Future Research Directions on Psychedelics and Positive Adult Development

There remains limited research on the relationship between naturalistic psychedelic use and positive or negative developmental outcomes for emerging adults. Positive impacts of psychedelic found in the literature use may be overstated due to self‐selection and confirmation biases (Bremler et al. 2023), while negative outcomes may be likely underreported (Evans et al. 2023). Because much of the existing research relies on cross‐sectional, self‐selected, and short‐term designs, the evidence base for developmental impacts remains preliminary and should be interpreted with caution. Although some longitudinal research has examined outcomes of psychedelic‐induced mystical experiences with follow‐ups of up to 6 months (Griffiths et al. 2011, 2008), most studies remain cross‐sectional and rely on uncontrolled observational designs (St. Arnaud and Sharpe, 2023b; Davis et al. 2022; Roseman et al. 2018; Amada et al. 2020). Developmental impact may occur over a much longer timescale and occurs in a much more organic manner than may be observed in a medical or therapeutic trial. Future research could use longitudinal studies with a cohort of individuals to demonstrate time‐series relationships between psychedelic use and adult developmental outcomes as well as retrospective reports of individuals who claimed to be positively impacted by psychedelic use during the period of emerging adulthood. A promising dataset may be the Monitoring the Future study, which has collected numerous cohorts of longitudinal data on emerging adults’ substance use and psychosocial developmental data since 1957 (Johnston et al. 2023). In terms of a bespoke longitudinal study on psychedelics and development in emerging adulthood, it would be ideal to use a range of measures that capture the aforementioned trajectories of adult development (see Supporting Information). These data would be analyzable to determine average trends, latent growth clusters, and individual time‐series cases.

For such future studies, additional moderators that could be examined include age of use, gender, socioeconomic status, personality factors, cultural background, and developmental history. There are many possible combinations of these contextual factors that may influence if the outcome of psychedelic use leads to a positive developmental impact. With regard to gender, emerging adult men consume psychedelics at a higher rate than women (AIHW 2024), possibly due to differences in risk‐taking (Harris and Jenkins 2006). Thus, due to men's relatively higher consumption, there may be important gender‐specific differences in motivations for psychedelic use and outcomes of psychedelic use on positive emerging adult development that could be examined in future studies. Likewise, another potentially important moderating factor is age of psychedelic use onset. Certain ages of psychedelic use onset during emerging adulthood may be more developmentally beneficial than others, such as later onset (25–29) being more beneficial than earlier (18–24), which is consistent with the harm profiles of other substances (Wisk and Weitzman 2016). Another potential moderating factor that warrants further research may be an emerging adult's attachment style (Cherniak et al. 2023; Cherniak et al. 2024). For example, in a naturalistic survey, Cherniak et al. (2024) found that perceptions of an insecure early attachment history were positively correlated with the intensity of psychedelic experiences. A potential source of vital information on prevalence/patterns of use, and associations, of psychedelic drugs in emerging adulthood is the Global Psychedelic Survey (University of Michigan 2025). This survey of thousands of adults around the world has the potential to provide important data on the prevalence of positive and adverse events after psychedelic experiences in emerging adulthood, compared to other age groups.

Another methodological challenge is the difficulty in defining what constitutes a positive developmental impact. As this review has highlighted, the developmental impacts of psychedelic use are likely to be complex, context dependent, and multidimensional. Qualitative and narrative research may be particularly well suited to capturing the paradoxical and dialectical nature of such long‐term changes, including the coexistence of challenge and growth reported by many users. Some psychedelic use may be both positive and negative, such as when individuals report a psychedelic experience to be both challenging and beneficial (Agin‐Liebes et al. 2024). Likewise, many constructs of positive adult development overlap empirically (Robinson 2020). For example, divergent thinking has been correlated with personality trait openness (McCrae 1987). As pointed out by Sjöström et al. (2024), personality trait openness predicts mental health (Lamers et al. 2012), and it is unknown whether individuals already with higher trait openness seek out psychedelics or if psychedelic use in fact increases trait openness. The present review used Robinson's (2020) framework as a broad tool to generate hypotheses, but future research is needed to operationalize different developmental trajectories and outcomes. For a list of potential measures for all the trajectories, see the Supporting Information.

Having reviewed and considered prospective opportunities for research in how psychedelics can support positive adult development in emerging adulthood, we now cover the associated risks. Given that this is not the principal focus of the article, we cover this in briefer terms as a critical counterpoint to the main thrust of our argument.

## 4 Risks of Psychedelic Use in Emerging Adulthood

Here, we consider the potential for adverse outcomes following psychedelic use and their potential to negatively impact developmental progress along the same developmental trajectories reviewed above. Ongoing brain development, potentially increased sensitivity to psychedelics (Aday et al. 2021), and generally higher trait risk‐taking (Terracciano et al. 2008) in emerging adulthood could lead to negative outcomes such as schizophrenia spectrum disorders (Hendin and Penn 2021; Cerón Tapia et al. 2022), hallucinogen‐use disorder, and HPPD, which are most prevalent in emerging adults, particularly males (APA 2022). While psychedelic use may facilitate development along some of Robinson's (2020) trajectories for certain emerging adults, it may hinder development for others. Further research is warranted to understand the risks of psychedelic use to emerging adult development described next, particularly what predicts whether development may be aided or hindered by psychedelic use.

### 4.1 Risks to Orthogenetic Development

As for orthogenetic development, there may be a risk of emerging adults becoming overwhelmed by psychedelic experiences and the dissolution of ego boundaries. This dissolution can lead to feelings of confusion, existential angst, and difficulty in integrating the psychedelic experience into one's sense of self (Winstock et al. 2014; Evans et al. 2023). It is conceivable that psychedelic experiences could overwhelm or destabilize emerging adults’ existing worldviews, leaving them struggling to integrate these experiences. In some cases, this difficulty might even lead to temporary regression in ego development, potentially hindering overall growth and maturation. One particular concern of note is psychedelic‐induced ego inflation (Hendin and Penn 2021; Masters and Houston 2000). This refers to users experiencing a magnified sense of self‐importance, grandiosity, and superiority—in extreme forms referred to as a “messianic complex” (Bowers and Freedman 1966). Speculatively, the heightened sensitivity and still‐maturing cognitive capacities characteristic of emerging adulthood could make individuals more prone to ego inflation. In such cases, psychedelic experiences might be misinterpreted as affirmations of exceptional insight or ability, temporarily bolstering a fragile and still‐forming sense of self. The effects of this phenomenon may resemble certain kinds of spiritual emergencies, which have been reported to be triggered by practices like meditation (St. Arnaud and Cormier 2017).

### 4.2 Risks to Eudaimonic Development

Within the eudaimonic development trajectory, it could be hypothesized that psychedelic use may at times hinder growth when emotional breakthroughs remain unresolved or insufficiently integrated. While psychedelics have the potential to bring suppressed emotions to the surface for conscious processing, emerging adults could struggle to effectively process and later integrate these intense emotional experiences. Moreover, some evidence suggests that a subset of psychedelic users may engage in use as a form of emotional avoidance or escape (Móró et al. 2011). Instead of confronting and working through their emotional challenges in a healthy manner, it is possible that they may turn compulsively to psychedelics to escape as in cases of hallucinogen‐use disorder (Evans et al. 2023). Additionally, psychedelics have been found to induce states of depersonalization and derealization (Evans et al. 2023), further disconnecting individuals from their emotions and inhibiting their ability to engage with their feelings. It could be hypothesized that these experiences may hinder the development of meaningful engagement with life and interpersonal connections. Furthermore, there is a hypothetical risk of emerging adults engaging in a phenomenon known as “spiritual bypassing,” which is a tendency to use “peak” or “transcendent” experiences to avoid facing unresolved emotional, psychological, developmental, interpersonal, vocational, physical, or other issues (Masters 2010). Future research should investigate if some emerging adults mistake transcendent experiences induced by psychedelics that felt personally significant for genuine self‐actualization that is objectively reflected in their day‐to‐day life.

### 4.3 Risks to Veridical‐Epistemic Development

In the domain of veridical‐epistemic development, one potential concern is the emergence of false or distorted psychedelic‐induced insights (McGovern et al. 2024; Timmermann et al. 2022). The valence of the outcome of PCIs is likely contingent on the credibility of the self‐insights gained (Letheby 2021). Thus, a challenge for emerging adults lies in discerning between truthful and untruthful PCIs, as any insight may seem profound or important during the experience but later prove false (McGovern et al. 2024). A risk is that if emerging adults integrate false PCIs into their day‐to‐day life, it may lead to negative outcomes in the future, such as regret and embarrassment. For example, a potential false insight related to the concept of “spiritual bypassing” may be the pre‐trans fallacy (Wilber 1982), which is a phenomenon where individuals confuse pre‐rational with trans‐rational stages of development. According to Wilber (1982), pre‐rational stages refer to earlier developmental levels characterized by concrete thinking, magical beliefs, and egocentrism, typical of childhood and early stages of human evolution (Wilber 1982). On the other hand, trans‐rational stages represent higher levels of development beyond conventional norms and structures, characterized by holistic thinking and true insight (Wilber 1982). Emerging adults’ potential misinterpretation of pre‐rational PCIs as indicative of trans‐rational wisdom can lead individuals to mistakenly view false insights gained during psychedelic experiences as profound truths, which if acted on may lead to negative developmental trajectories.

### 4.4 Risks to Relational Development

As for relational development, psychedelic use may pose a risk to emerging adults’ ability to cultivate and maintain meaningful connections with others. It has been found that one significant risk of psychedelic use is social disconnection, where individuals may become isolated in their experiences following psychedelic use (Evans et al. 2023). Although empirical research on social consequences of psychedelic use in emerging adulthood is scarce, it could be hypothesized that new insights, personality shifts, or changes in spirituality following psychedelic experiences might lead some individuals to feel disconnected from their pre‐existing social circles, including friends and romantic partners. Qualitative reports from adult and clinical samples suggest that psychedelic experiences can alter personal values and social priorities (e.g., Carhart‐Harris et al. 2018; Nayak and Griffiths 2022), but the developmental implications of such shifts remain largely unexplored. Future research could investigate whether changes in worldview or identity following psychedelic use are associated with measurable differences in social connectedness, perceived belonging, or relational satisfaction among emerging adults. A program of research addressing these possibilities might combine longitudinal and qualitative approaches to track changes in social network composition and meaning‐making over time. It might also test whether difficulties articulating ineffable psychedelic experiences predict later feelings of alienation or misunderstanding in close relationships. Finally, researchers could examine whether greater emphasis on introspective or spiritual growth following psychedelic use corresponds with reduced engagement in everyday interpersonal relationships.

### 4.5 Risks to Ethical Development

The role of psychedelic use in shaping ethical development during emerging adulthood remains largely unexplored and warrants careful examination. Grof (1988) has proposed that psychedelics may act as “nonspecific amplifiers” that accelerate individuals’ latent processes and direction of development (Grof 1988). From this perspective, psychedelics may be limited in their ability to encourage ethical behavior in a particular direction and instead just accelerate its current direction. As highlighted by Pace and Devenot (2021), psychedelics can also be used by individuals who espouse fringe or extremist ideologies (e.g., Nazism) and may not necessarily lead to changes in ethical outlook or behavior. Thus, psychedelics may not guarantee a shift toward social concern and nature relatedness (Noorani et al. 2018; Schmid and Liechti 2018). Furthermore, as discussed above, emerging adults risk their behavior being inspired by false PCIs that may at the time have had good intentions but in hindsight reflect on their behavior as ethically neutral or unethical.

## 5 Conclusion

Emerging adulthood is a transitional period of human development, where orthogenetic, veridical‐epistemic, eudaimonic, relational, and ethical development continue and interact. Although some preliminary empirical findings and conceptual discussions suggest that psychedelic use may relate to these domains of emerging adult development, systematic empirical research across these areas remains sparse. Although limited research so far has focused on psychedelic use among emerging adults specifically, our review outlines a number of theoretical links supporting potential developmental impacts. Future well‐controlled studies are required to further elucidate the nuanced ways in which psychedelic use may both support and hinder development during emerging adulthood.

## Author Contributions

J.P. conceptualized the paper, performed the majority of the literature search, and wrote most of the paper. O.R., K.S.A., J.S.A., and M.W. wrote parts of the paper and contributed to drafts. C.L. and G.M. provided critical oversight of the paper.

## Funding

The authors have nothing to report.

## Conflicts of Interest

The authors declare no conflicts of interest.

## Peer Review

The peer review history for this article is available at https://doi.org/10.1002/brb3.71043.

## Supporting information




**Supplementary Material**: brb371043‐sup‐0001‐SuppMat.docx

## Data Availability

Data sharing not applicable to this article as no datasets were generated or analysed during the current study.
