# Supplementary material for: The Relationship Between Psychedelic Use and Positive Adult Development in Emerging Adulthood: An Integrative Review
Source: Brain Behav. 2025 Nov 14;15(11):e71043. doi: 10.1002/brb3.71043 (PMC12617272; doi:10.1002/brb3.71043)
Supplement: Supplementary file 1 — Supplementary Material: brb371043‐sup‐0001‐SuppMat.docx [file BRB3-15-e71043-s001.docx]

**Supplementary Material**

**The Relationship Between Psychedelic Use and Positive Adult Development in Emerging Adulthood: An Integrative Review**

**Potential Measures of Orthogenetic Development**

· Titles and Realistic Problem Generation Test

o Runco, M. A., Dow, G., & Smith, W. R. (2006). Information, experience, divergent thinking: An empirical test. Creativity Research Journal, 18, 269-277.

· Aesthetic Emotions Scale

o Schindler, I., Hosoya, G., Menninghaus, W., Beermann, U., Wagner, V., Eid, M., & Scherer, K. R. (2017). Measuring aesthetic emotions: A review of the literature and a new assessment tool. PloS one, 12(6), e0178899.

· Quiet Ego Scale

o Wayment, H. A., Bauer, J. J., & Sylaska, K. (2015). The quiet ego scale: measuring the compassionate self-identity. Journal of Happiness Studies, 16, 999-1033.

· Subject-Object Interview

o Lahey, L., Felix, S., Goodman, R., Kegan, R., & Souvaine, E. (2011). A guide to the subject-object interview: Its administration and interpretation. Minds at Work.

· Reflective Judgment Interview (RJI)

o King, P. M., & Kitchener, K. S. (2004). Reflective judgment: Theory and research on the development of epistemic assumptions through adulthood. Educational psychologist, 39(1), 5-18.

**Potential Measures of Eudaimonic Development**

· The Life Satisfaction Scale

o Diener, E., Emmons, R. A., Larsen, R. J., & Griffin, S. (1985). The Satisfaction With Life Scale. Journal of Personality Assessment, 49(1), 71–75. https://doi.org/10.1207/s15327752jpa4901_13 Wellbeing

· The Warwick-Edinburgh Mental Well-being Scale

o Tennant, R., Hiller, L., Fishwick, R., Platt, S., Joseph, S., Weich, S., & Stewart-Brown, S. (2007). The Warwick-Edinburgh mental well-being scale S-5 (WEMWBS): development and UK validation. Health and Quality of life Outcomes, 5(1), 1-13. doi:10.1186/1477-7525-5-63

· The Meaning in Life Questionnaire

o Steger, M. F., Frazier, P., Oishi, S., & Kaler, M. (2006). The meaning in life questionnaire: assessing the presence of and search for meaning in life. Journal of counseling psychology, 53(1), 80.

· Difficulties in Emotion Regulation Scale

o Gratz KL, Roemer L (2004) Multidimensional assessment of emotion regulation and dysregulation: Development, factor structure, and initial validation of the difficulties in emotion regulation scale. J Psychopathol Behav Assess 26: 41–54.

· The Questionnaire for Eudaimonic Well-Being

o Waterman AS, Schwartz SJ, Zamboanga BL, Ravert RD, Williams MK, Agocha MB, Donnellan VB: The Questionnaire for Eudaimonic Well-Being: Psychometric properties, demographic comparisons, and evidence of validity. The Journal of Positive Psychology 2010,5(1):41–61. doi:10.1080/17439760903435208 doi:10.1080/17439760903435208

· Self-Determination Interview (SDI)

o Shogren, K. A., Little, T. D., Grandfield, E., Raley, S., Wehmeyer, M. L., Lang, K. M., & Shaw, L. A. (2020). The Self-Determination Inventory–Student Report: Confirming the factor structure of a new measure. Assessment for Effective Intervention, 45(2), 110-120.

**Potential Measures of Veridical-Epistemic Development**

· Defense Mechanisms Rating Scales Q-Sort

o Di Giuseppe M, Perry JC. The Hierarchy of Defense Mechanisms: Assessing Defensive Functioning With the Defense Mechanisms Rating Scales Q-Sort. Front Psychol. 2021 Oct 15;12:718440. doi: 10.3389/fpsyg.2021.718440. PMID: 34721167; PMCID: PMC8555762.

· The Self-Reflection and Insight Scale

o Grant, A. M., Franklin, J., & Langford, P. (2002). The self-reflection and insight scale: A new measure of private self-consciousness. Social Behavior and Personality: an international journal, 30(8), 821-835.

· Integrative Self-Knowledge Scale

o Ghorbani, N., Watson, P. J., & Hargis, M. B. (2008). Integrative Self-Knowledge Scale: Correlations and Incremental Validity of a Cross-Cultural Measure Developed in Iran and the United States. The Journal of Psychology, 142(4), 395–412.<https://doi.org/10.3200/JRPL.142.4.395-412>

· Three-Dimensional Wisdom Scale (3D-WS)

o Wang, I. Y., & Cheung, R. Y. (2025). Three-Dimensional Wisdom Scale (3D-WS). In Handbook of Assessment in Mindfulness Research (pp. 1967-1976). Cham: Springer Nature Switzerland.

**Potential Measures of Relational-Reproductive Development**

· Relationship Assessment Scale

o Hendrick, S. S. (1988). A generic measure of relationship satisfaction. Journal of Marriage and the Family, 93-98.

· Satisfaction with Relationship Status Scale (ReSta)

o Lehmann, V., Tuinman, M. A., Braeken, J., Vingerhoets, A. J., Sanderman, R., & Hagedoorn, M. (2015). Satisfaction with relationship status: Development of a new scale and the role in predicting well-being. Journal of happiness studies, 16, 169-184.

· The Romantic Partner Conflict Scale

o Zacchilli, T. L., Hendrick, C., & Hendrick, S. S. (2009). The romantic partner conflict scale: A new scale to measure relationship conflict. Journal of Social and Personal Relationships, 26(8), 1073-1096.

· Adult Attachment Interview (AAI)

o George, C., Main, M., & Kaplan, N. (1996). Adult attachment interview. Interpersona: An International Journal on Personal Relationships.

**Potential Measures of Ethical Development**

· Ten Item Value Inventory

o Sandy, C. J., Gosling, S. D., Schwartz, S. H., & Koelkebeck, T. (2017). The development and validation of brief and ultrabrief measures of values. Journal of personality assessment, 99(5), 545-555

· Emotional Empathic Tendency Scale

o Mehrabian, A. 1 994. Manual for the Emotional Empathic Tendency Scale(EETS). Available from Albert Mehrabian, 1 1 30 Alta Mesa Road, Monterey, CA 93940.

· Nature Relatedness Scale

o Nisbet, E. K., & Zelenski, J. M. (2013). The NR-6: A new brief measure of nature relatedness. Frontiers in psychology, 4, 813.

· Moral Judgment Interview (MJI)

o Gibbs, J. C., Widaman, K. F., & Colby, A. (1982). Construction and validation of a simplified, group-administerable equivalent to the moral judgment interview. Child development, 895-910.
